# Integrated 16S rRNA sequencing and nontargeted metabolomics analysis to reveal the mechanisms of Yu-Ye Tang on type 2 diabetes mellitus rats

**DOI:** 10.3389/fendo.2023.1159707

**Published:** 2023-09-05

**Authors:** Ziang Ma, Wenjuan Sun, Lixin Wang, Yuansong Wang, Baochao Pan, Xiuhai Su, Hanzhou Li, Hui Zhang, Shuquan Lv, Hongwu Wang

**Affiliations:** ^1^ Graduate School of Hebei University of Chinese Medicine, Shijiazhuang, China; ^2^ Cangzhou Hospital of Integrated Traditional Chinese Medicine and Western Medicine of Hebei Province Affiliated to Hebei University of Chinese Medicine, Cangzhou, China; ^3^ College of Integrated Chinese and Western Medicine, Tianjin University of Traditional Chinese Medicine, Tianjin, China; ^4^ College of Traditional Chinese Medicine, Tianjin University of Traditional Chinese Medicine, Tianjin, China

**Keywords:** type 2 diabetes mellitus, Yu-Ye Tang, gut microbiota, tryptophan metabolism, glycerophospholipid metabolism

## Abstract

**Introduction:**

Yu–Ye Tang (YYT) is a classical formula widely used in treatment of type 2 diabetes mellitus (T2DM). However, the specific mechanism of YYT in treating T2DM is not clear.

**Methods:**

The aim of this study was to investigate the therapeutic effect of YYT on T2DM by establishing a rat model of T2DM. The mechanism of action of YYT was also explored through investigating gut microbiota and serum metabolites.

**Results:**

The results indicated YYT had significant therapeutic effects on T2DM. Moreover, YYT could increase the abundance of *Lactobacillus, Candidatus_Saccharimonas, UCG-005, Bacteroides* and *Blautia* while decrease the abundance of and *Allobaculum* and *Desulfovibrio* in gut microbiota of T2DM rats. Nontargeted metabolomics analysis showed YYT treatment could regulate arachidonic acid metabolism, alanine, aspartate and glutamate metabolism, arginine and proline metabolism, glycerophospholipid metabolism, pentose and glucuronate interconversions, phenylalanine metabolism, steroid hormone biosynthesis, terpenoid backbone biosynthesis, tryptophan metabolism, and tyrosine metabolism in T2DM rats.

**Discussion:**

In conclusion, our research showed that YYT has a wide range of therapeutic effects on T2DM rats, including antioxidative and anti-inflammatory effects. Furthermore, YYT corrected the altered gut microbiota and serum metabolites in T2DM rats. This study suggests that YYT may have a therapeutic impact on T2DM by regulating gut microbiota and modulating tryptophan and glycerophospholipid metabolism, which are potential key pathways in treating T2DM.

## Introduction

Type 2 diabetes mellitus (T2DM) is a prevalent form of diabetes that affects more than 90% of individuals diagnosed with the diabetes (1). T2DM can occur at any age and has an insidious onset with no early manifestations. Therefore, it is necessary to have a well-planned and effective medication plan to achieve desirable glycemic regulation. If the blood glucose level is not controlled in a timely manner, the disease may affect several organs and cause complications, such as eye and foot problems, as the duration of the disease increases and reduce the patients’ quality of life (2). As a typical chronic disease, long-term medication is the most important means to control the blood glucose level. Despite the availability of a wide range of glucose-lowering drugs in clinical practice, only approximately 50% of patients with diabetes achieve their glycemic control goals. Therefore, effective prevention and treatment of T2DM has become the focus of social concern.

The gut microbiota creates a complex ecosystem in gastrointestinal tract that helps maintain the energy balance of the body and good health (3). An increasing number of studies have revealed significant role that gut microbiota plays in human health and its imbalances have relationships with various diseases, including diabetes, fatty liver, and obesity (4, 5). A previous study showed that fecal transplantation from patients with obesity into germ-free mice resulted in weight gain in mice (6). In addition, a study found the abundance of *Akkermansia muciniphila* was reduced in T2DM patients and was positively correlated with reduced insulin secretion (7). These results suggest that gut microbiota is important in T2DM progression by regulating metabolic processes. It is a novel idea for T2DM treatment through regulating gut microbiota.

Many natural compounds had been found to ameliorate diabetes through regulating the gut microbiota. Astragaloside IV, an active ingredient *of Astragalus propinquus*, increases the level of butyric acid in gut microbiota of T2DM mice and exerts therapeutic effects on T2DM by regulating AMPK/SIRT1 and PI3K/AKT signaling pathways (8). Pi–Dan–Jian–Qing decoction can ameliorate liver and kidney function and reduce inflammation and oxidative stress in T2DM rats, which may be associated with increasing abundance of probiotics at genus level (9). Gegen–Qinlian decoction showed to reduce blood glucose levels and protect islet function in rats with T2DM by altering the gut microbiota composition. Specifically, this effect is achieved by increasing the proportion of anti-inflammatory short-chain fatty acids producing bacteria and decreasing proportion of bacteria associated with a diabetic phenotype and potential pathogenic properties (10).

Yu–Ye Tang (YYT) is a classical decoction widely used to treat T2DM. It is consisted of *Dioscorea oppositifolia* L., *Astragalus mongholicus* Bunge, *Anemarrhena asphodeloides* Bunge, chicken’s gizzard membrane, *Pueraria montana* var. *lobata*, *Schisandra chinensis*, and *Trichosanthes rosthornii*. *In vivo* studies have shown that YYT could ameliorate liver and kidney injury in T2DM rats (11). Besides, accumulated studies have revealed potential mechanisms of active compounds in YYT. The main active component of *Dioscorea oppositifolia* L. includes allantoin, which has been demonstrated with protective effects on pancreas β-cells in metabolic dysfunction model (12). Allantoin can also improve nonalcoholic steatohepatitis disease by activating the SIRT1/Nrf2 pathway, increasing the activities of antioxidant enzymes, and reducing lipid peroxidation (13). Astragaloside is the main component of *Astragalus mongholicus* Bunge, and several studies have confirmed that astragaloside could reduce inflammation and oxidative stress in animal model of diabetes (14, 15). Timosaponin B-II is the main steroidal saponin component of *Anemarrhena asphodeloides* Bunge, which has shown with antioxidative and anti-inflammatory effects on hyperglycemia (16, 17). Puerarin, an isoflavone from *Pueraria montana* var. *lobata*, mainly acts on PI3K-Akt, tumor necrosis factor (TNF) and reactive oxygen species related pathways to exert anti-inflammatory and antioxidative effects (18–20). Schisandrin, isolated from *Schisandra chinensis*, has been shown to improve diabetes-related pathologies and has a wide range of effects in terms of antioxidant, anti-inflammation, and immune modulation (21, 22). However, specific mechanism of YYT in treating T2DM is not well understood. To better understand this, the present study established a T2DM rat model and used two approaches to study the effects of YYT on T2DM. First, we examined the therapeutic effects of YYT on T2DM. Second, we used 16s rRNA gene sequencing and nontargeted metabolomics to explore the mechanism by which YYT acts to treat T2DM.

## Methods

### YYT preparation

Mix 30 g of *Dioscorea oppositifolia* L., 15 g of *Astragalus mongholicus* Bunge, 18 g of *Anemarrhena asphodeloides* Bunge, 6 g of chicken’s gizzard membrane, 5 g of *Pueraria montana* var. *lobata*, 9 g of *Schisandra chinensis*, and 9g of *Trichosanthes rosthornii*. The ingredients were first pulverized and sieved, then mixed with water, soaked for 0.5 hours, and boiled for 2 hours. The mixture was then filtered, and the process was repeated with a second batch of water. The two decoctions were combined, and the solution was then concentrated to a concentration of 5 kg crude herb/L on a thermostatic water bath.

### Reagents

Streptozotocin (STZ, S8050) was purchased from Solarbio Biotechnology (Beijing, China). Metformin (MET, S30880) was purchased from Yuanye Biotechnology (Shanghai, China). Biochemical test kits of total cholesterol (TC), triglyceride (TG), alanine aminotransferase (ALT), aspartate aminotransferase (AST), creatinine (Cr), blood urea nitrogen (BUN), superoxide dismutase (SOD), glutathione peroxidase (GSH-Px), and methane dicarboxylic aldehyde (MDA) were purchased from Nanjing Jiancheng Biological Engineering Institute (Nanjing, China). Enzyme-linked immunosorbent assay (ELISA) kits for insulin (mlE2721), interleukin (IL)-1β, IL-6, and TNF-α were purchased from Shanghai Enzyme-linked Biotechnology (Shanghai, China).

### Animals

Sixty SPF-grade healthy male SD rats (180–220 g) were purchased from HFK Bio-Technology and housed in ventilated environment (20°C–26°C, 50%–60% relative humidity) with *ad libitum*. The experiment was approved by the Ethics Committee of Cangzhou Integrated Traditional Chinese and Western Medicine Hospital (No. CZX2021-KY-026).

### Generation of T2DM rat model

A high-sugar, high-fat diet (HSHFD, 42.3% of carbohydrate, 17% of protein, 22.5% of fat, 3.2% of fiber, 5% of minerals, 10% of moisture) was administered for 8 weeks. Subsequently, 30 mg/kg of STZ was injected intraperitoneally after 12 h of fasting food. Meanwhile, rats in control group received normal diet (60% of carbohydrate, 16% of protein, 3% of fat, 4% of fiber, 5% of minerals, 12% of moisture) and vehicle. Seventy-two hours after the injection, randomly blood glucose was measured, and the level of blood glucose ≥16.7 mmol/L was considered as the T2DM model generation criteria.

### Animal experiment

The rats were acclimatized for 1 week, and then divided into the control group, model group, metformin (MET) group, YYT low-dose (YYT-L) group, YYT medium-dose (YYT-M) group, and YYT high-dose (YYT-H) group randomly. After modeling, the MET group was intragastrically administered with MET 0.2 g/kg/d, and the YYT-L, YYT-M and YYT-H groups were intragastrically administered with YYT 4.1g/kg/d, 8.2g/kg/d, and 16.4g/kg/d. The control and model groups were intragastrically administered with 0.5 mL/d of vehicle. All groups were treated for 4 consecutive weeks, and fasting blood glucose (FBG) of each rat was measured weekly. All therapeutic doses were calculated using the pharmacological body surface area method. The doses of YYT-M and MET groups corresponded to the equivalent dose for 70 kg adult humans, while the doses of YYT-L and YYT-H groups represented 0.5-fold and 2-fold M-HDP group doses, respectively.

**Figure 1:**
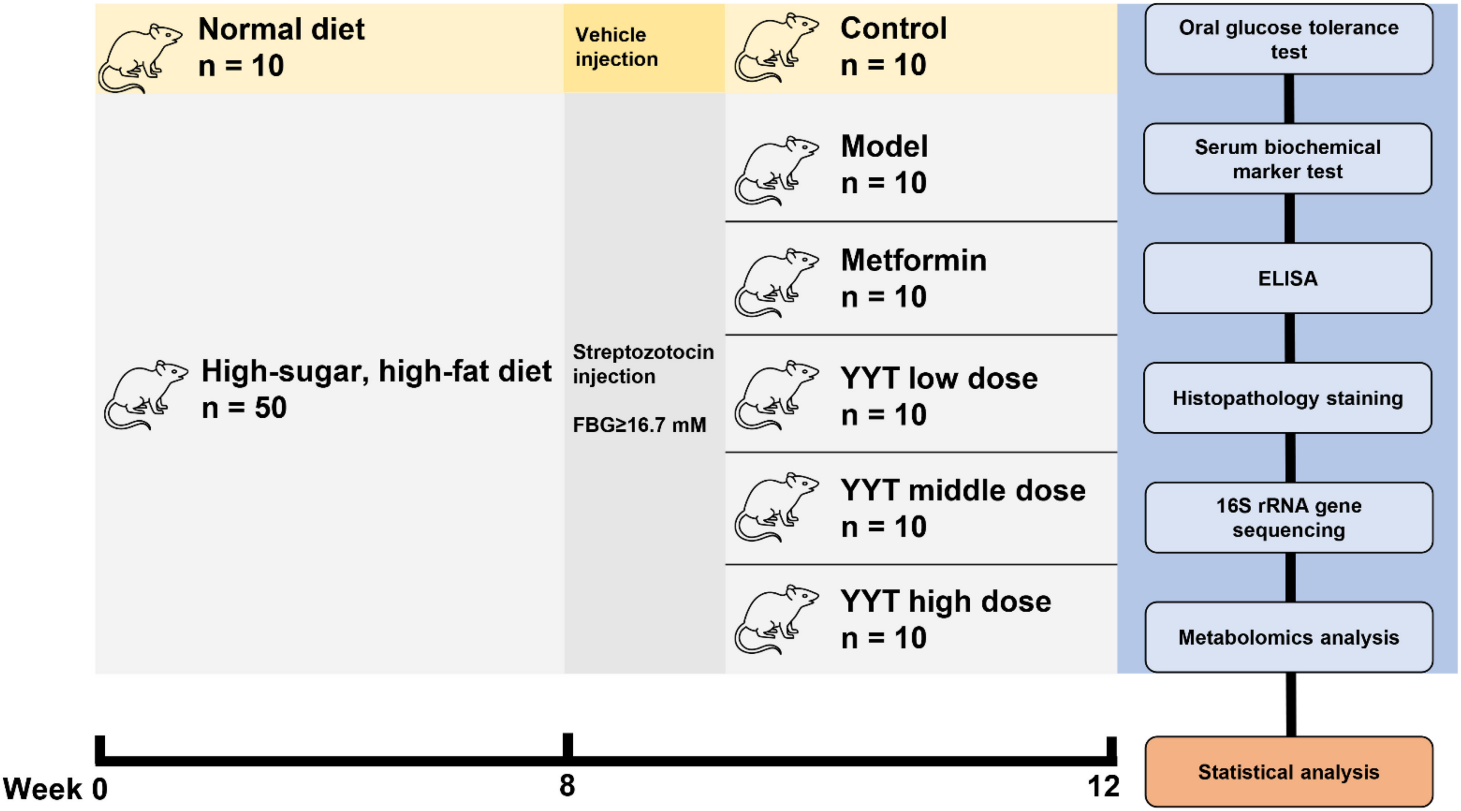
Schematic diagram.

After a 4-week treatment period, an oral glucose tolerance test (OGTT) was carried out. The rats were then fasted for 12 hours and anesthetized. Blood was taken from the abdominal aorta, and the rats were then euthanized. Tissues from the liver, pancreas, and kidney were collected and preserved in 4% paraformaldehyde. Feces was also collected from the cecum (**Figure 1**).

### OGTT

First, the rats were fasted without water for 12 hours. Blood glucose levels were measured using glucose testing strips. These levels were recorded as the 0-minute blood glucose values. Next, a 50% glucose solution (2 g/kg) was given intragastrically, and the blood glucose levels were measured at 15, 30, 60, and 120 minutes after administration. The results were recorded. The data collected from the OGTT was plotted on a blood glucose-time curve, and the area under the curve (AUC) was calculated.

### Serum biochemical marker test

The blood collected from abdominal aorta was centrifuged (3,000 rpm, 10 min), and the serum was collected. Assay kits were used to detect the levels of TG, TC, ALT, AST, C, BUN, and MDA. Serum SOD, GSH-PX activities were also tested. All protocols were carried out according to the instructions provided by the manufacturer.

### ELISA

Serum levels of fasting insulin (FINS), IL-6, IL-1β, and TNF-α were measured using ELISA, which was performed based on manufacturers’ instructions. The homeostatic model assessment for insulin resistance (HOMA-IR) was calculated with formula: HOMA-IR = (FBG × FINS)/22.5.

### Histopathology staining

The liver, pancreas, and kidney that had been fixed were processed by being dehydrated and embedded in paraffin. Afterwards, 5 micrometer thick sections were cut from the paraffin blocks. After deparaffinization, routine hematoxylin and eosin (HE) staining was performed, and histopathological changes were observed under a light microscope. Histopathogical changes in liver, kidney, and pancreas were assessed and scored as previous described (23–25). For liver injury scoring, the degree was assessed as follows: 0 (normal); 1 (development of a sinusoidal congestion space); 2–3 (the presence and/or severity of sinusoidal congestion and cytoplasmic vacuolization); and 4 (necrosis of parenchymal cells and hemorrhage). For kidney injury scoring, the degree was assessed by the area of pathological change area as follows: 0 (none), 1 (≤ 10%), 2 (11-25%), 3 (26-45%), 4 (46-75%), and 5 (≥ 76%). For pancreas injury scoring, the area of pancreas occupied by the islets of Langerhans were evaluated.

### 16S rRNA gene sequencing

Two hundred mg of fecal samples were weighed and the genomic DNA of each sample was extracted using the cetyltrimethylammonium bromide method. The quality and concentration of the extracted genomic DNA was examined. The quality was assessed using 1% agarose gel electrophoresis and the concentration was measured. The DNA was then diluted to a final concentration of 1 ng/µL. The detailed protocols of polymerase chain reaction amplification, sequencing data processing and analysis were involved in **Supplementary Material**.

### Metabolomics analysis

The serum sample was prepared as follows: 100 μL of serum was mixed with 400 μL of 80% methanol, vortexed, shaken, and chilled. The mixture was then centrifuged at 4°C (15,000 g, 20 min). The supernatant was diluted with ultrapure water to 53% methanol and centrifuged again at 4°C (15,000 g, 20 min). The resulting supernatant was collected and used as the sample for testing. Detailed protocols of quality control, liquid chromatography–mass spectrometry, and data processing and analysis were involved in **Supplementary Material**.

### Statistical analysis

Statistical analysis was performed using SPSS software. The experimental data were expressed as mean ± standard deviation (SD), and one-way analysis of variance with Tukey’s honestly significant difference test was used. Spearman’s correlation analysis was used to evaluate the correlation of therapeutic indicators and changed gut microbiota. A difference with *p* < 0.05 was considered to be statistically significant. The graphs were developed by GraphPad Prism 9.

## Results

### Therapeutic effect of YYT on T2DM rats

**Figure 2:**
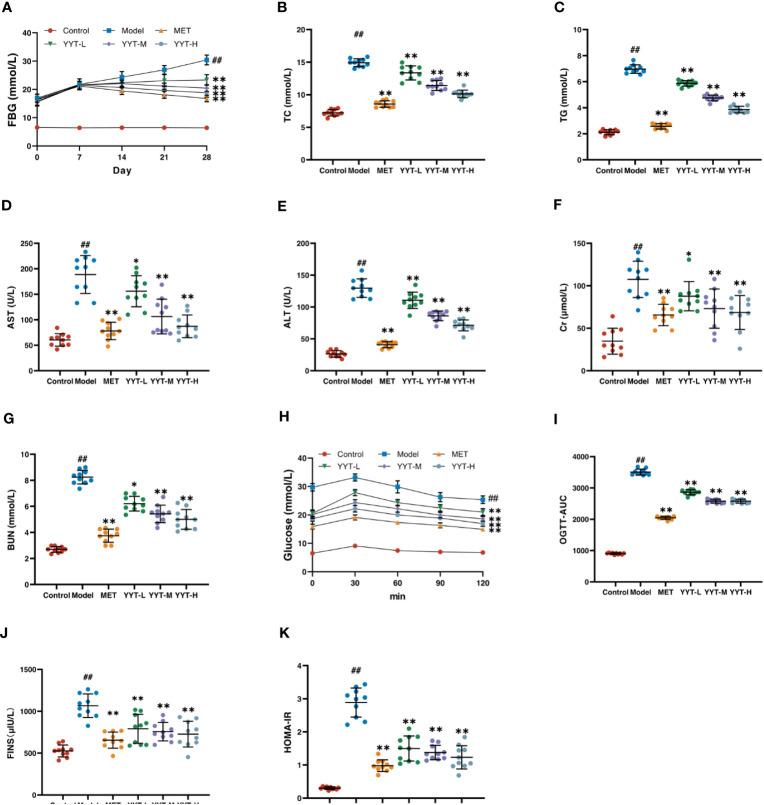
YYT had therapeutic effects on model rats. **(A)** FBG was controlled after YYT treatment. **(B–G)** The elevated physiological indices of blood lipid, liver and kidney functions were improved after YYT treatment. **(H, I)** OGTT showed the impaired glucose tolerance was ameliorated after YYT treatment. **(J, K)** FINS and HOMA-IR showed YYT could reduce the increased IR. Control, model, MET, YYT-L, YYT-M and YYT-H (n = 10 per group) groups. ##: *p* < 0.01 *vs*. control group. *: *p* < 0.05 and **: *p* < 0.01 *vs*. model group.

The changes in FBG level of rats in each group were observed weekly from the beginning of drug administration. The FBG levels were elevated in model group compared with control group, and the level was reduced in the MET, YYT-L, YYT-M, and YYT-H groups after 2, 3, and 4 weeks of drug administration compared with the model group (**Figure 2A**) After the treatment, serum levels of TC, TG, AST, ALT, Cr, and BUN were increased in T2DM rats compared with those in control group. Compared with the model group, TC, TG, AST, ALT, Cr, and BUN levels were decreased in MET, YYT-L, YYT-M, and YYT-H groups, which suggesting that YYT has a positive effect on blood lipids, liver function, and kidney function in T2DM rats (**Figures 2B–G**).

OGTT was performed for all rats after the treatment. The results showed that in all groups, blood glucose levels peaked at 30 minutes after oral glucose administration, before gradually declining. At 120 minutes after administration, the OGTT and OGTT-AUC were higher in model group compared to control group. However, OGTT and OGTT-AUC were reduced in the MET, YYT-L, YYT-M, and YYT-H groups when compared to model group (**Figures 2H, I**). Furthermore, the results showed a significant increase in FINS and HOMA-IR levels in model group compared to control group, indicating presence of insulin resistance (IR) in T2DM rats. Conversely, the MET, YYT-L, YYT-M, and YYT-H groups had significantly reduced FINS and HOMA-IR levels compared to model group (**Figures 2J, K**). These results suggest that YYT can reduce the level of IR in T2DM rats.

**Figure 3:**
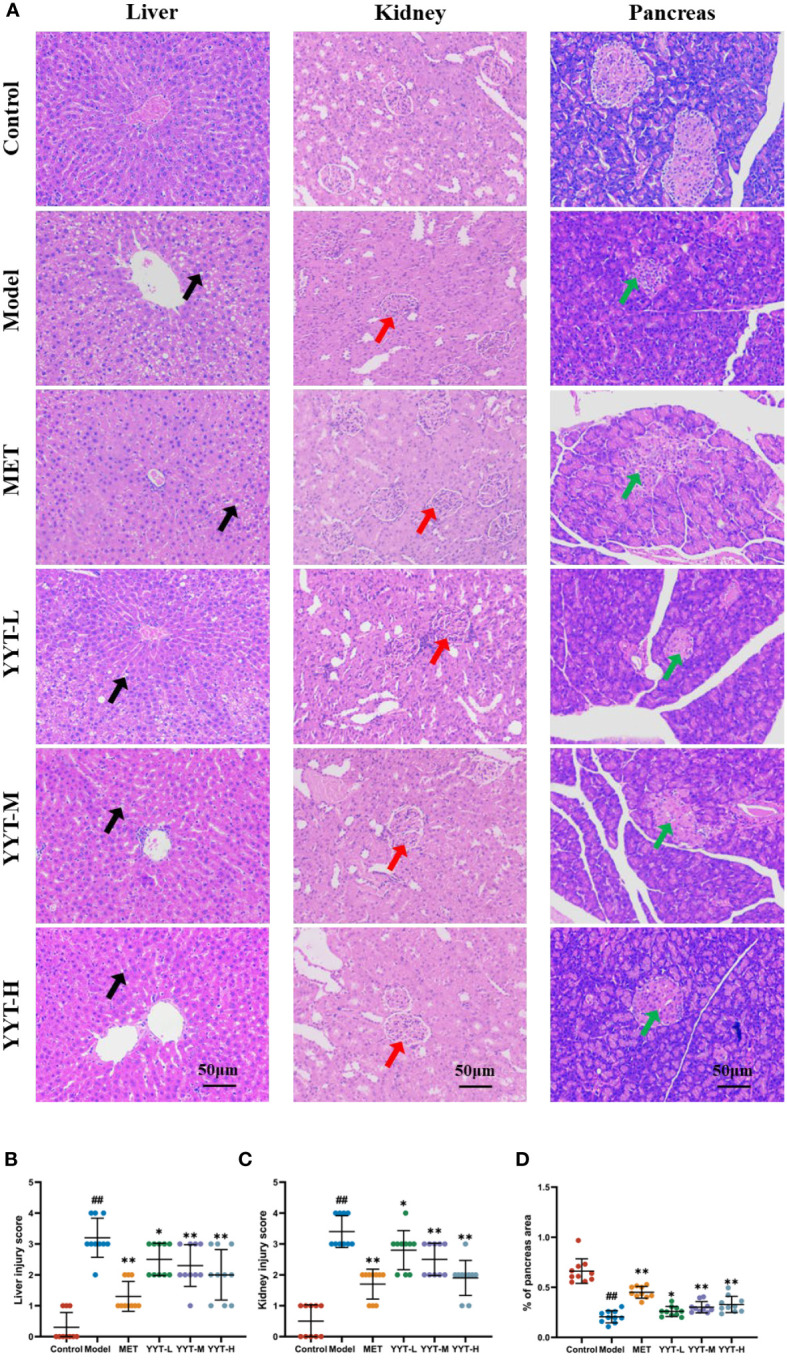
**(A)** HE staining showed that the pathological changes in the damaged liver, kidney, and pancreas of T2DM rats were improved after receiving YYT treatment. **(B–D)** The histopathogical quantification results also showed the treatment of YYT could ameliorate the injury in liver, kidney, and pancreas. The black arrow indicated hepatocyte vacuolation and steatosis. The red arrow indicated damaged glomerular. The green arrow indicated islets with irregular morphology, blurred borders, and disorganized structure. ##: *p* < 0.01 *vs*. control group. *: *p* < 0.05 and **: *p* < 0.01 *vs*. model group.

HE staining of liver showed that the hepatic cords in control group were neatly arranged, with no significant inflammatory cell infiltration and hepatocyte vacuolation and steatosis. On the contrary, the hepatic cords of rats in model group were disordered, with more inflammatory cell infiltration and a large number of hepatocytes vacuolation and steatosis. The hepatic cords of each treatment group were neatly arranged after treatment, with a small amount of inflammatory cell infiltration and scattered hepatocyte vacuolation and steatosis seen. HE staining results of kidney showed that glomerular structure was clear and intact in the control group, with no increase in the mesangial matrix and no inflammatory cell infiltration. The results showed that compared to control group, the glomeruli in model group were significantly hypertrophic, with increased mesangial matrix, vacuolated tubular epithelial cells, and interstitial infiltration with inflammatory cells. However, the kidney of rats in each intervention group exhibited varying degrees of improvement in the above pathology compared with model group. Mild glomerular mesangial hyperplasia was noted, the renal tubular structure was nearly normal, and not much inflammatory cell infiltration was seen in the interstitium. HE staining of pancreatic tissues showed that the islets of rats in the control group had regular structure, clear edges, abundant cells, uniform size, saturated morphology, and tight arrangement. Moreover, no vacuoles or significant pathological changes were found. In the model group, the islets of rats had irregular morphology, blurred borders, disorganized structure, and deformed cells. Compared with T2DM rats, number of cells in islets of rats in each treatment group increased significantly, with regular structure and no significant necrosis. The islets had clear borders and improved morphology and structure (**Figure 3A**). Likewise, quantitative analysis of pathological results indicated that YYT treatment lowered the pathological scores of liver, kidney and pancreas (**Figures 3B–D**).

### Antioxidative and anti-inflammatory effects of YYT on T2DM rats

**Figure 4:**
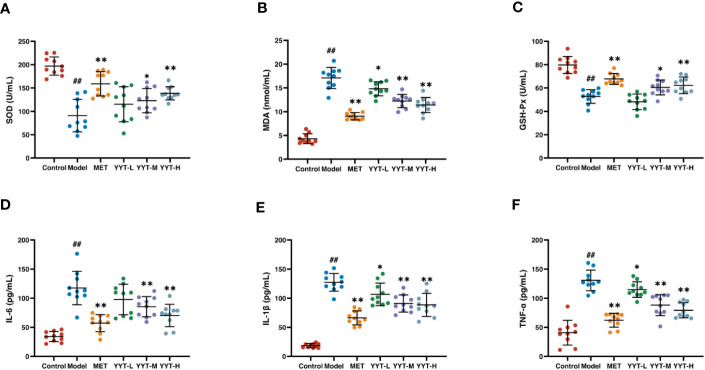
YYT treatment had antioxidative and anti-inflammatory effects on T2DM rats. **(A–C)** YYT could increase the lowered activities SOD and GSH-Px while decrease the level of MDA in serum of T2DM rats. **(D–F)** The elevated serum levels of pro-inflammatory cytokines (IL-6, IL-1β, TNF-α) in T2DM rats could be ameliorated by YYT. Control, model, MET, YYT-L, YYT-M and YYT-H (n = 10 per group) groups. ##: *p* < 0.01 *vs.* control group. *: *p* < 0.05 and **: *p* < 0.01 *vs.* model group.

The T2DM rats showed significant decreases in activities of SOD and GSH-PX and an increase in MDA concentration in serum compared to control group, indicating that T2DM caused oxidative stress damage to the rats. In contrast, the rats in the MET, YYT-L, YYT-M, and YYT-H groups showed increases in activities of SOD and GSH-PX and decreases in MDA concentration compared to T2DM model group (**Figures 4A–C**). This result shows that YYT treatment significantly reduces oxidative stress injury in T2DM rats.

ELISA results indicated that, in comparison to control group, levels of IL-6, IL-1β, and TNF-α were significantly elevated in model group, which suggests that T2DM leads to an increase in pro-inflammatory cytokines. However, when compared to model group, levels of all 3 cytokines were significantly decreased in the MET, YYT-L, YYT-M, and YYT-H groups, indicating that the administration of the herbal decoction was effective in reducing the levels of pro-inflammatory cytokines in T2DM rats (**Figures 4D–F**).

The above results revealed that YYT-H has significant therapeutic effects on T2DM rats. Therefore, 16S rRNA gene sequencing and metabolomic studies were performed in control, model, and YYT-H groups.

### Effect of YYT on the gut microbiota of T2DM rats

**Figure 5:**
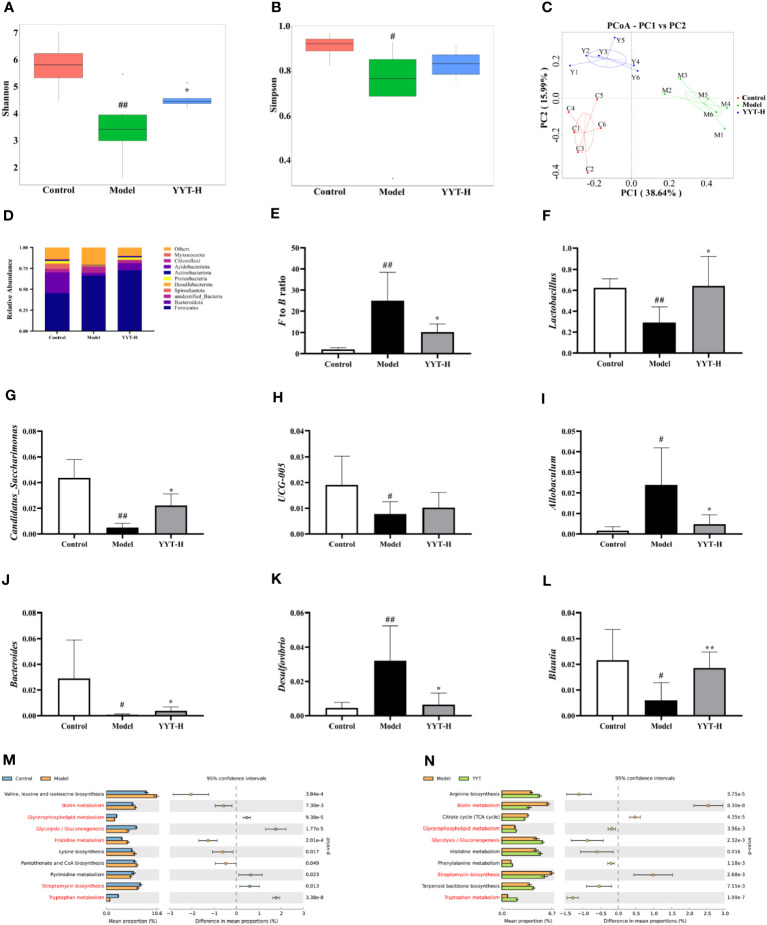
YYT modulates gut microbiota in rats altered by T2DM **(A, B)** YYT treatment decreased Shannon and Simpson indexes in T2DM rats. **(C)** Score plot of PCoA showed diversity of gut microbiota in each group. **(D–F)** Gut microbiota at phylum level was dominated by *Firmicutes* and *Bacteroidota* and the F/B ratio was decreased in T2DM rats after YYT treatment. **(F–L)** Relative abundances of *Lactobacillus, Candidatus_Saccharimonas, UCG-005, Allobaculum, Bacteroides, Desulfovibrio* and *Blautia* were mostly affected in T2DM rats or after YYT treatment. **(M, N)** PICRUSt analysis based on the results of genus levels of gut microbiota. Common pathways between two comparisons were marked in red. Control, model, and YYT-H (n = 6 per group) groups. #: *p* < 0.05 and ##: *p* < 0.01 *vs*. control group. *: *p* < 0.05 and **: *p* < 0.01, *vs*. model group.

The α diversity analysis was performed to evaluate the species diversity of the gut microbiota in each group. The analysis was based on the Shannon and Simpson indexes, which provide insight into the richness and evenness of species in a sample. Results showed that compared to control group, both indexes were reduced in model group, indicating a decrease in species diversity. However, when comparing model group to YYT-H group, both indexes increased, suggesting that YYT-H had the ability to enhance the richness and evenness of gut microbiota in rats (**Figures 5A, B**). The β-diversity analysis in this study was performed to compare the microbial community structure between different samples. The principal co-ordinates analysis (PCoA) method was used to assess the similarity or difference in community composition. **Figure 5C** shows that samples from different treatments are represented by different colors or dots. The closer the distance between two sample dots, the greater the similarity in their microbial composition. The gut microbiota in the control and model groups were separated, indicating significant changes in the gut microbiome of the T2DM rat model. After YYT-H intervention, the gut microbiota appeared to shift closer to the control group, suggesting that YYT-H had a regulatory effect on the community structure of the gut microbiome in rats.

The analysis of gut microbiota composition showed that rat gut microbiota at phylum level was dominated by *Firmicutes* and *Bacteroidota* (**Figure 5D**). The results showed that the *Firmicutes*/*Bacteroidota* (F/B) ratio was significantly higher in model group compared to control group. After treatment with YYT-H, the ratio decreased, suggesting a modulating effect of YYT-H on the gut microbiota composition (**Figure 5E**). *Lactobacillus, Candidatus_Saccharimonas, UCG-005, Allobaculum, Bacteroides, Desulfovibrio*, and *Blautia* were the dominant genera of rat gut microbiota in each group. Compared with control group, *Allobaculum* and *Desulfovibrio* were increased and *Lactobacillus, Candidatus_Saccharimonas, Bacteroides*, and *Blautia* were significantly decreased in model group. Compared with model group, *Lactobacillus, Candidatus_Saccharimonas, UCG-005, Bacteroides* and *Blautia* were increased and *Allobaculum* and *Desulfovibrio* were significantly decreased after YYT treatment (**Figures 5F–L**).

Analysis of gut microbiota function through PICRUSt using genus levels of gut microbiota showed differences among control, model, and YYT-H groups. The top 10 metabolic pathways showed differences between the groups are presented in **Figures 5M, N**. The results indicate that YYT-H intervention had an impact on the metabolic processes in T2DM rats.

### Effect of YYT on serum metabolites in T2DM rats

**Figure 6:**
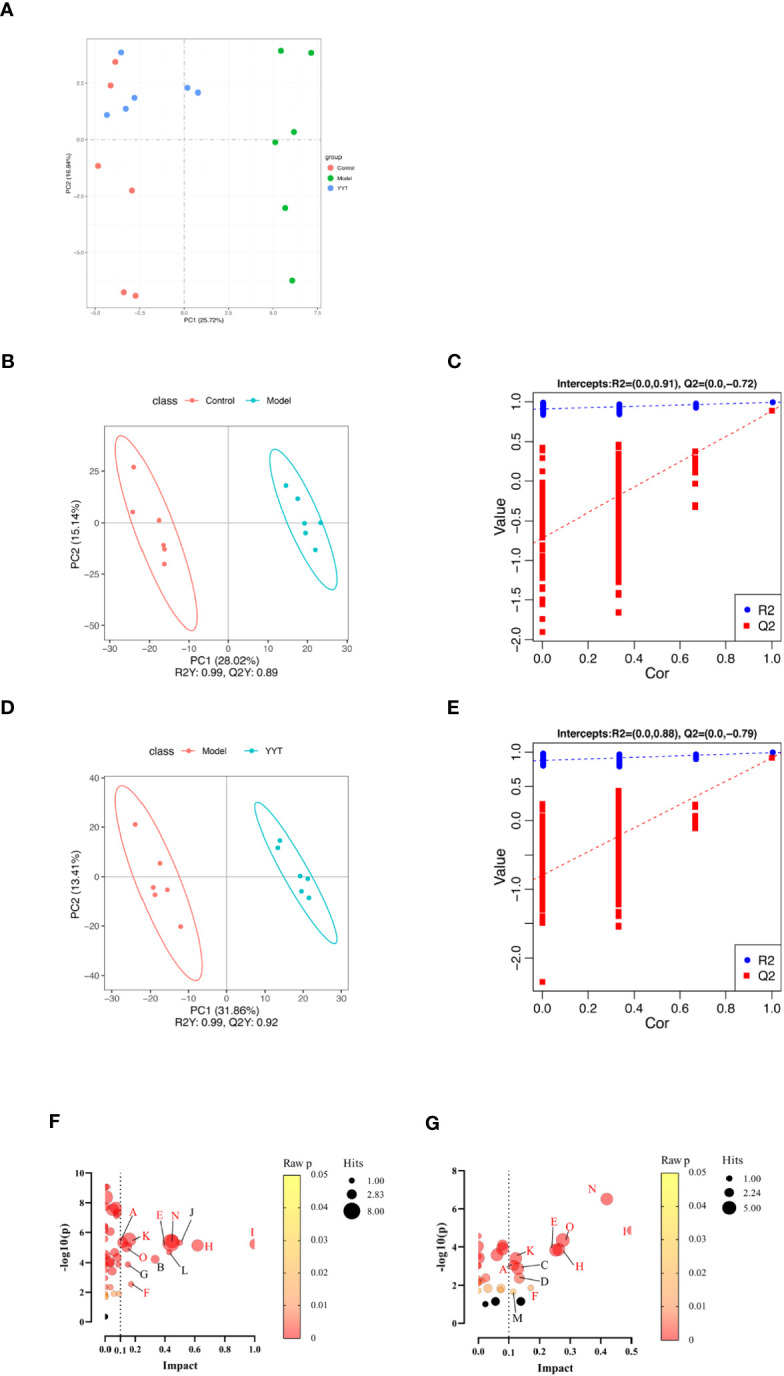
Changes in serum metabolites of T2DM rats after YYT treatment. **(A)** PCA score showed that there was a clear separation between control and model groups and between model and YYT groups. **(B–E)** Scores plots of PLS-DA and corresponding coefficient of loading plots. **(F, G)** Name of pathway. a: Alanine, aspartate and glutamate metabolism, b: alpha-Linolenic acid metabolism, c: Arachidonic acid metabolism, d: Arginine and proline metabolism, e: Glycerophospholipid metabolism, f: Pentose and glucuronate interconversions, g: Pentose phosphate pathway, h: Phenylalanine metabolism, i: Phenylalanine, tyrosine and tryptophan biosynthesis, j: Riboflavin metabolism, k: Steroid hormone biosynthesis, l: Taurine and hypotaurine metabolism, m: Terpenoid backbone biosynthesis, n: Tryptophan metabolism, o: Tyrosine metabolism. Black bubbles mean *p* ≥ 0.05. The common pathways between two comparisons were written in red. Control, model, and YYT-H (n = 6 per group) groups.

The results of the principal components analysis (PCA) on the serum metabolites of control, model and YYT-H groups are shown in **Figure 6A**. The score plot indicates that the serum metabolic profile of T2DM rats was altered compared to control group, as evidenced by the clear separation in the two-dimensional space. Additionally, the score plot for the model group and YYT-H group shows that the serum metabolic profile of the YYT-H group was also altered, as demonstrated by a clear separation in the two-dimensional space. Additionally, YYT-H group was closer to control group than model group, indicating that YYT could ameliorate the altered serum metabolites caused by T2DM. A partial least squares-discriminant analysis (PLS-DA) model was developed to further evaluate the explanation and prediction capability of the serum samples. This model was designed to produce better separation and reduce any confounding factors (**Figures 6B–E**). The results showed that the serum metabolic profiles had good separation between control and model groups, as well as between model and YYT-H groups. The coefficient of determination (R2Y) was 0.99 and the goodness-of-fit (Q2Y) was 0.89 for the comparison between control and model groups, and R2Y was 0.99 and Q2Y was 0.92 for the comparison between model and YYT-H groups, indicating that the model had a high explanatory power. To ensure that the model wasn’t overfitting, a cross-validation experiment was performed, and the results showed that the goodness-of-fit was negative, suggesting that the model had a good predictive power.

The following three criteria were used to screen for differential metabolites: fold change >1.2, *p* < 0.05 and variable importance in projection (VIP) > 1.0. The differential metabolites were then screened by KEGG pathway analysis and a total of 67 differential metabolites related with metabolic pathways were obtained from the screening (**Table 1**). Differential pathways were screened according to impact value > 0.1 and *p* < 0.05. The screened pathways between control and model groups included alanine, aspartate and glutamate metabolism, alpha-Linolenic acid metabolism, glycerophospholipid metabolism, pentose phosphate pathway, pentose and glucuronate interconversions, phenylalanine, tyrosine and tryptophan biosynthesis, phenylalanine metabolism, riboflavin metabolism, steroid hormone biosynthesis, taurine and hypotaurine metabolism, tryptophan metabolism, and tyrosine metabolism (**Figure 6F**). Between model and YYT groups, alanine, aspartate and glutamate metabolism, arginine and proline metabolism, arachidonic acid metabolism, glycerophospholipid metabolism, pentose and glucuronate interconversions, phenylalanine metabolism, steroid hormone biosynthesis, terpenoid backbone biosynthesis, tryptophan metabolism, and tyrosine metabolism were the differential pathways (**Figure 6G**). Among these, tryptophan metabolism and glycerophospholipid metabolism were the common pathways between the prediction of PICRUSt analysis and pathway analysis of nontargeted metabolomics.

**Table 1:** The differential metabolites in serum related with pathways.

No.	Formula	RT	m/z	Metabolites	VIP		FC		Trend		Pathway
		(min)			M vs. C	Y vs. M	M vs. C	Y vs. M	M vs. C	Y vs. M	
1	C_26_H_52_NO_7_P	14.89	520.34	1-Oleoyl-Sn-Glycero-3-Phosphocholine	2.00	1.79	0.47	1.86	↓##	↑**	E
2	C_7_H_7_NO_3_	3.31	154.05	3-Hydroxyanthranilic acid	1.34	1.31	0.48	1.79	↓	↑*	N
3	C_11_H_12_N_2_O_3_	6.55	221.09	5-Hydroxytryptophan	1.24	2.23	0.62	2.07	↓#	↑**	N
4	C_3_H_7_N_3_O_2_	1.31	118.06	Guanidineacetic acid	2.37	1.92	0.38	2.10	↓##	↑**	D
5	C_5_H_10_N_2_O_3_	1.32	147.08	L-Glutamine	1.80	1.85	0.61	1.63	↓##	↑**	A
6	C_10_H_12_N_2_O_3_	8.39	209.09	L-Kynurenine	1.48	1.95	0.17	8.01	↓##	↑**	N
7	C_22_H_46_NO_7_P	14.69	468.31	LPC 14:0	1.61	1.71	0.59	1.69	↓##	↑**	E
8	C_24_H_48_NO_7_P	14.37	538.31	LPC 16:1	1.55	1.21	0.29	2.50	↓#	↑*	E
9	C_26_H_52_NO_7_P	14.77	566.35	LPC 18:1	1.97	2.04	0.56	1.72	↓##	↑**	E
10	C_28_H_52_NO_7_P	14.72	604.36	LPC 20:3	1.84	2.16	0.31	3.65	↓##	↑**	E
11	C_28_H_50_NO_7_P	14.49	588.33	LPC 20:4	2.35	1.53	0.28	2.16	↓##	↑**	E
12	C_28_H_48_NO_7_P	14.09	600.33	LPC 20:5	1.95	1.66	0.29	2.98	↓##	↑*	E
13	C_30_H_54_NO_7_P	14.92	630.38	LPC 22:4	1.99	1.50	0.34	2.22	↓##	↑**	E
14	C_30_H_52_NO_7_P	14.63	628.36	LPC 22:5	1.87	1.39	0.30	2.42	↓##	↑**	E
15	C_11_H_12_N_2_O_2_	6.70	205.10	L-Tryptophan	1.54	1.08	0.52	1.51	↓#	↑*	N
16	C_9_H_11_NO_3_	1.98	180.07	L-Tyrosine	2.23	2.19	0.38	2.47	↓##	↑**	I, O
17	C_22_H_46_NO_7_P	13.72	466.30	Lysopc 14:0	1.41	1.29	0.40	2.25	↓##	↑*	E
18	C_28_H_50_NO_7_P	14.49	542.32	Lysopc 20:4	2.34	1.67	0.29	2.30	↓##	↑**	E
19	C_42_H_82_NO_7_P	15.04	744.59	PC (14:0e/20:2)	1.11	1.52	1.67	0.52	↑##	↓**	E
20	C_42_H_80_NO_7_P	15.95	742.58	PC (14:1e/20:2)	1.61	1.74	1.94	0.46	↑##	↓**	E
21	C_39_H_78_NO_8_P	15.03	720.55	PC (15:0/16:0)	1.21	1.27	1.60	0.57	↑	↓**	E
22	C_46_H_82_NO_8_P	14.87	866.59	PC (18:1/20:4)	1.61	1.94	0.38	2.99	↓##	↑**	E
23	C_44_H_80_NO_7_P	15.78	766.58	PC (18:1e/18:4)	1.25	1.27	1.67	0.58	↑#	↓**	E
24	C_9_H_8_O_3_	1.98	163.04	Phenylpyruvic acid	2.19	1.87	0.48	1.81	↓##	↑**	H, I
25	C_19_H_28_O_2_	12.49	289.22	Testosterone	1.11	1.78	0.48	3.44	↓#	↑*	K
26	C_5_H_12_O_5_	1.33	151.06	Xylitol	1.10	1.20	1.85	0.54	↑#	↓*	F
27	C_44_H_80_NO_8_P	16.68	840.58	PC (16:0/20:4)	0.81	1.46	0.67	1.88	↓	↑*	E
28	C_46_H_84_NO_8_P	16.17	868.61	PC (18:0/20:4)	1.01	1.35	0.52	1.74	↓	↑**	E
29	C_46_H_82_NO_8_P	13.87	808.58	PC (18:0/20:5)	1.04	1.68	0.47	2.80	↓#	↑**	E
30	C_48_H_86_NO_8_P	16.44	894.62	PC (18:0/22:5)	0.89	1.10	0.67	1.44	↓	↑*	E
31	C_48_H_82_NO_8_P	16.35	890.59	PC (20:3/20:4)	0.65	1.84	0.62	2.64	↓	↑**	E
32	C_28_H_58_NO_7_P	15.57	610.40	LPC 20:0	1.26	1.48	0.69	1.48	↓	↑*	E
33	C_30_H_62_NO_7_P	16.51	638.44	LPC 22:0	0.68	1.24	1.24	0.65	↑	↓**	E
34	C_19_H_39_O_7_P	14.04	409.24	LPA 16:0	0.11	1.01	0.92	0.68	↓	↓*	E
35	C_9_H_11_NO_4_	6.69	198.08	Levodopa	1.54	2.00	0.64	1.83	↓#	↑*	O
36	C_4_H_4_O_4_	1.20	115.00	Fumaric acid	0.58	1.31	1.33	0.54	↑	↓*	A, O
37	C_21_H_32_O_3_	13.16	331.23	17α-Hydroxypregnenolone	0.80	1.05	1.93	0.35	↑	↓*	K
38	C_21_H_30_O_5_	11.14	363.22	Cortisol	0.70	1.47	0.66	2.30	↓	↑*	K
39	C_20_H_32_O_6_	13.13	369.23	Prostaglandin G2	0.56	1.51	1.88	0.15	↑	↓*	C
40	C_30_H_47_N_3_O_9_S	12.81	626.31	Leukotriene C4	0.91	1.83	2.56	0.15	↑	↓*	C
41	C_5_H_12_N_2_O_2_	1.14	133.10	Ornithine	1.03	1.27	0.44	2.29	↓#	↑*	D
42	C_6_H_12_O_4_	1.34	147.07	Mevalonic acid	0.33	1.24	1.15	0.60	↑	↓*	M
43	C_9_H_11_NO_2_	4.54	166.09	L-Phenylalanine	1.16	0.23	0.82	1.02	↓#	↑	H, I
44	C_17_H_20_N_4_O_6_	7.92	377.15	Vitamin B2	1.36	0.72	0.46	1.34	↓#	↑	J
45	C_43_H_76_NO_8_P	16.38	766.54	PE (18:0/20:5)	1.30	0.15	1.61	1.06	↑#	↑	E
46	C_5_H_14_NO_4_P	14.43	184.07	Phosphocholine	1.14	0.75	0.81	1.14	↓##	↑*	E
47	C_5_H_13_NO	13.51	104.11	Choline	1.20	0.60	0.46	1.39	↓##	↑	E
48	C_44_H_82_NO_8_P	15.64	860.60	PC (16:0/20:3)	1.24	1.27	0.64	1.53	↓#	↑	E
49	C_44_H_82_NO_8_P	15.40	784.58	PC (18:1/18:2)	1.13	0.58	1.60	1.33	↑#	↑	E
50	C_42_H_84_NO_7_P	15.62	746.61	PC (18:1e/16:0)	1.24	0.12	1.42	1.00	↑#	↑	E
51	C_24_H_50_NO_7_P	14.76	540.33	LPC 16:0	1.72	0.26	0.56	0.97	↓##	↓	E
52	C_26_H_54_NO_7_P	15.29	582.38	LPC 18:0	1.44	0.32	0.70	1.06	↓##	↑	E
53	C_26_H_50_NO_7_P	14.55	564.33	LPC 18:2	2.04	0.77	0.40	1.41	↓##	↑	E
54	C_26_H_48_NO_7_P	14.28	576.33	LPC 18:3	1.35	1.08	0.44	2.03	↓##	↑	E
55	C_30_H_50_NO_7_P	14.37	568.34	LPC 22:6	1.47	0.88	0.43	1.63	↓##	↑**	E
56	C_25_H_52_NO_7_P	15.29	508.34	Lysopc 17:0	1.57	0.64	0.67	1.12	↓##	↑	E
57	C_26_H_54_NO_7_P	15.29	522.36	LysoPC 18:0	1.82	0.24	0.65	1.05	↓##	↑	E
58	C_26_H_50_NO_7_P	14.42	518.33	Lysopc 18:2	1.60	0.59	0.41	1.49	↓##	↑	E
59	C_8_H_20_NO_6_P	1.42	258.11	Choline Glycerophosphate	1.21	0.52	0.59	1.19	↓#	↑	E
60	C_21_H_39_O_7_P	14.98	433.24	LPA 18:2	1.63	1.38	0.58	1.59	↓##	↑	E
61	C_2_H_7_NO_3_S	1.24	124.01	Taurine	1.19	0.88	0.53	1.47	↓#	↑	L
62	C_18_H_30_O_2_	14.76	279.23	α-Linolenic acid	1.29	0.82	2.40	0.60	↑##	↓	B
63	C_21_H_30_O_3_	11.49	331.23	17alpha-Hydroxyprogesterone	1.60	1.01	2.49	0.57	↑#	↓*	K
64	C_21_H_28_O_5_	11.25	361.20	Cortisone	1.72	0.95	2.04	0.65	↑#	↓*	K
65	C_18_H_22_O_3_	12.83	287.16	16α-Hydroxyestrone	2.46	0.89	6.02	0.44	↑##	↓*	K
66	C_9_H_10_O_4_	2.09	181.05	Homovanillic acid	1.20	0.89	0.66	1.36	↓#	↑	O
67	C_4_H_6_O_6_	15.20	149.01	L-(+)-Tartaric acid	1.20	0.98	0.72	1.31	↓#	↑	G

Control (C), model (M) and YYT-H (Y) groups (n = 6 per group). #: *p* < 0.05 and ##: *p* < 0.01 *vs*. control group. *: p < 0.05, and **: p < 0.01 vs. model group.

A: Alanine, aspartate and glutamate metabolism, B: alpha-Linolenic acid metabolism, C: Arachidonic acid metabolism, D: Arginine and proline metabolism, E: Glycerophospholipid metabolism, F: Pentose and glucuronate interconversions, G: Pentose phosphate pathway, H: Phenylalanine metabolism, I: Phenylalanine, tyrosine and tryptophan biosynthesis, J: Riboflavin metabolism, K: Steroid hormone biosynthesis, L: Taurine and hypotaurine metabolism, M: Terpenoid backbone biosynthesis, N: Tryptophan metabolism, O: Tyrosine metabolism

**Figure 7:**
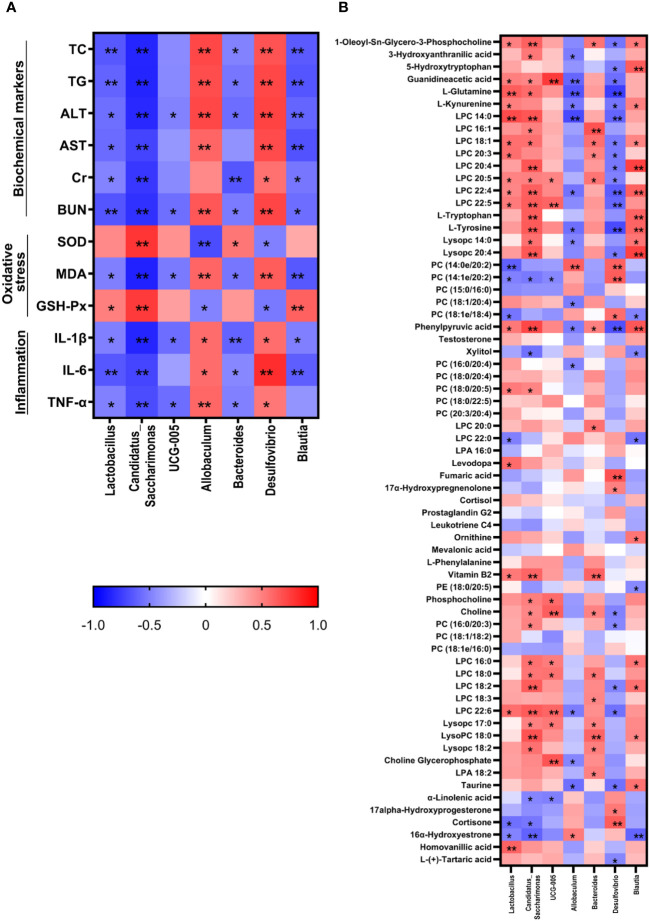
Correlation analysis between therapeutic indicators **(A)** and altered gut microbiota **(B)** was performed using Spearman's analysis and visualized as a heatmap. The color-coding scale in the heatmap represents the Pearson correlation coefficient, where deeper red or blue shades indicate higher absolute values of the coefficient. *: *P* < 0.05, **: *P* < 0.01.

The correlation analysis showed that *Lactobacillus, Candidatus_Saccharimonas, UCG-005, Bacteroides*, and *Blautia* were negatively correlated with most therapeutic indicators while *Allobaculum* and *Desulfovibrio* showed positive correlation with most of the therapeutic indicators (**Figure 7**). Additionally, *Candidatus_Saccharimonas* and *Blautia* were positive with most differential metabolites while *Allobaculum* and *Desulfovibrio* were negative with most differential metabolites.

## Discussion

In this study, we used HSHFD combined with STZ injection to construct the T2DM rat model. This method is commonly used to induce T2DM in rats. HSHFD could cause IR and small dose (30mg/kg) of STZ injection (normally more than 100mg/kg of STZ was used to induce T1DM model) could damage part of the β‐cell of pancreas. These were similar as the pathological features of T2DM patients (26). In comparison to the control group, T2DM rats exhibited notable elevations in levels of blood glucose and lipid contents, along with irregularities in biochemical markers associated with liver and kidney functions. Moreover, OGTT-AUC and HOMA-IR values were also heightened, indicating the presence of IR. The pathological results revealed that the hepatic cords in liver of T2DM rats were disorganized, with more inflammatory cell infiltration, a large number of hepatocyte vacuoles, and fatty degeneration; the glomeruli in kidney were significantly hypertrophied, with increased mesangial matrix, vacuolar degeneration of renal tubular epithelial cells, and interstitial infiltration with inflammatory cells. The pancreatic islets in the pancreatic tissue had irregular morphology, blurred borders, disorganized structure, and deformed cells, which is consistent with the pathological manifestations of T2DM (27–29). The results of this study showed that YYT intervention reduced blood glucose and lipid levels, ameliorated levels of biochemical markers related to liver and kidney function, and alleviated histopathological changes in the liver, kidney, and pancreas of T2DM rats. The most significant effect was observed in YYT-H group, indicating that YYT has a therapeutic effect on T2DM. In addition, we selected metformin as the positive control drug (30, 31). Our results showed less differences in controlling blood glucose, reducing IR, and improving the biochemical markers between high-dose YYT and metformin treatment. However, more studies could be carried out to compare the effects of YYT and metformin on different stages of T2DM in clinic. Besides, clinical studies could also be carried out to evaluate the therapeutic effects of combination of YYT and metformin on T2DM clinically.

The prolonged state of hyperglycemia and hyperlipidemia leads to a decrease in efficacy of antioxidant enzymes, which generates oxidative stress, hinders cellular and tissue functionality, exacerbates the disruption of normal glucose metabolism, and exacerbates the onset and progression of diabetes (32, 33). Therefore, it is equally important to measure the antioxidant capacity of the drug in T2DM rats. Our results indicated that YYT treatment elevated levels of the antioxidant markers SOD and GSH-PX in serum of T2DM rats while decreasing MDA levels. SOD operates as an antioxidant by eliminating superoxide anions, while GSH-PX protects cells and tissues by breaking down harmful peroxides. MDA, a product of oxidative stress, serves as an indicator of the extent of oxidative harm inflicted on cells (34, 35). The results also indicate that YYT effectively reduced levels of serum pro-inflammatory cytokines, such as IL-1β, IL-6, and TNF-α, in T2DM rats. These cytokines play a central role in regulating the inflammatory and immune response and participate in a wide range of physiological and pathological processes. Excessive production of these cytokines has been linked to the development of IR in various tissues, including adipose tissue, muscle, and liver leading to increased local inflammation and contributing to systemic IR and β-cell damage (36) IL-1 family of cytokines are critical regulators of immune and inflammatory response (37). IL-1β is particularly harmful as it is a major mediator of inflammatory response (38, 39). Research has shown that IL-6, a crucial member of the cytokine family, is a key factor in the development of IR (40). TNF-α is also known to induce IR (41). Elevated levels of inflammatory mediators, particularly IL-1β and TNF-α, exacerbate local inflammation, which can contribute to systemic IR and β-cell injury (42–44).

The gut microbiota plays an important role in development and progression of T2DM (45). The diversity of gut microbiota facilitates the maintenance of essential amino acid production, glucose metabolism, and the regulation of immune function (46, 47). Some studies have shown that adopting a healthy dietary strategy in prediabetes can help probiotic growth and prevent development of T2DM (48). The composition of the gut microbiota in many diabetes patients is altered, resulting in dysbiosis or even the loss of certain species. This leads to changes in the composition of fecal metabolites, which can cause inflammation, oxidative stress, and IR (49, 50). *Firmicutes* (gram-positive bacteria) and *Bacteroidota* (gram-negative bacteria) make up more than 90% of the total bacterial population in humans, and the F/B ratio is positively correlated with serum glucose levels. Antibiotic therapy improves insulin activity and increases glucose tolerance after lowering the *Firmicutes* levels (51). Therefore, correcting and maintaining the gut microbiota diversity is important in improving T2DM. The results of the study indicate that YYT has an improvement effect on gut microbiota, which may be one of the mechanisms by which it aids in the treatment of T2DM.

*Lactobacillus* is a widely recognized probiotic, and several species of the genus have been shown to lower and control blood glucose levels, enhance antioxidant capacity, exert anti-inflammatory effect, and prevent organ damage (52–54). *Candidatus_Saccharimonas* is an acid-producing bacterium that has an important role in maintaining intestinal pH (55). *Ruminococcaceae UCG-005* can enhance the production of short-chain fatty acids, which in turn regulates the expression of Bax and Bcl-2 in islets, thereby alleviating islet damage (56, 57). *Allobaculum* is positively correlated with the lipid metabolism regulator ANGPTL4, and significantly elevated relative abundance was detected in intestine and feces of HFD-induced obese mice, suggesting that it is intimately associated with lipid metabolism (58). Moreover, its relative abundance showed a positive correlation with blood glucose level in T2DM rats (59). The abundance of *Bacteroides* in patients with T2DM is often half of that in normal individuals, and *Ganoderma lucidum* polysaccharides can enhance the abundance of *Bacteroide*s in patients with T2DM (60, 61). *Desulfovibrio* is a group of sulfate-reducing bacteria in which hydrogen sulfide is a specific metabolite that activates the AKT signaling pathway, thereby improving IR (62). However, studies have found that patients with T2DM tend to have a higher abundance of *Desulfovibrio*. Hence, it has also been suggested that an increase in sulfate-reducing bacteria is an intrinsic aspect of T2DM pathogenesis. These contradictions imply that more studies are required to determine the role of this bacterium in T2DM (63, 64). The genus *Blautia* includes various acetate and butyrate producers (65, 66). It was observed that *Blautia* abundance was decreased in patients with T2DM compared with healthy individuals (67). Oral *Blautia* administration significantly improved metabolic disorders, including obesity and diabetes mellitus (68).

Matching the differential metabolic pathways obtained from metabolomics with those predicted using 16s rRNA gene sequencing revealed that tryptophan and glycerophospholipid metabolism are the common metabolic pathways. These may be the potential mechanisms by which YYT exerts its effects on treating T2DM through the regulation of the gut microbiota.

Tryptophan metabolism and its related metabolites are closely related to sugar intake and insulin sensitivity (69, 70). In the present study, the levels of L-tryptophan, L-kynurenine, and 5-hydroxytryptophan (5-HT) were significantly decreased in T2DM model rats, whereas the above metabolites and 3-hydroxyanthranilic acid (3-HAA) were significantly increased after YYT treatment. In rats, L-tryptophan enhances glucose-related energy expenditure and, thus, suppresses blood glucose elevation (71). Most of tryptophan catabolism occurs via the kynurenine synthesis pathway. L-kynurenine is an important intermediate in the synthesis pathway of kynurenine. Previous animal studies have demonstrated that reduced levels of tryptophan and kynurenine occur in both pre-diabetes and post-diabetes and are potential biomarkers of prediabetes (72). This phenomenon may be related to chronic inflammation, which upregulates the activity of tryptophan 2,3-dioxygenase and indoleamine 2,3-dioxygenase, the rate-limiting enzymes in the conversion of tryptophan to kynurenine (73). 3-HAA, one of the downstream products of L-kynurenine, exhibits anti-inflammatory and lipid-regulating functions (74, 75). 5-HT is one of the intermediates of the serotonin metabolic pathway, and its conversion to 5-hydroxyindoleacetic acid is accelerated when serum tryptophan levels are reduced in patients with T2DM, which may contribute to the reduced levels of 5-HT (76).

Glycerophospholipid metabolism is considered a significant pathway in T2DM in the context of lipid metabolism, and this pathway has been shown to be a key pathway for some drugs used to treat T2DM (77, 78). The levels of phosphatidylcholine (PC), lysoPC, phosphatidate, choline, phosphocholine, and choline glycerophosphate were reduced in T2DM rats compared with those of control group. After YYT treatment, PC and lysoPC levels were elevated. is a type of glycerophospholipid that is an important component of cell membranes. Abnormalities in hepatic PC synthesis have been observed during the development of diabetes and are thought to be associated with mitochondrial dysfunction (79). In addition, one study found that a high intake of PC decreased the occurrence of T2DM in a male population (80). This suggests that glycerophospholipid metabolism, including PC synthesis, may play a role in the development of T2DM. However, relationship between PC metabolism and T2DM remains to be further investigated. LysoPC has been shown to have potential benefits in management of diabetes, including improvement of IR, increased uptake of glucose by adipocytes, and potential benefits in both T1DM and T2DM (81, 82). It has been found that lysoPC could alleviate fatty acid-induced muscle cell injury through activation of PPARδ (83). However, research has also found that lysoPC levels are elevated in diabetic mice, which may promote IR (84). The specific mechanism of action of lysoPCs remains an open question.

There are some limitations to this study. The detailed relationship between differential metabolites and gut microbiota still required to be studied. Fecal transplantation and targeted metabolomics may be useful in future to deeply illustrate the metabolic regulatory mechanism of YYT on T2DM based on regulating gut microbiota.

## Conclusion

**Figure 8:**
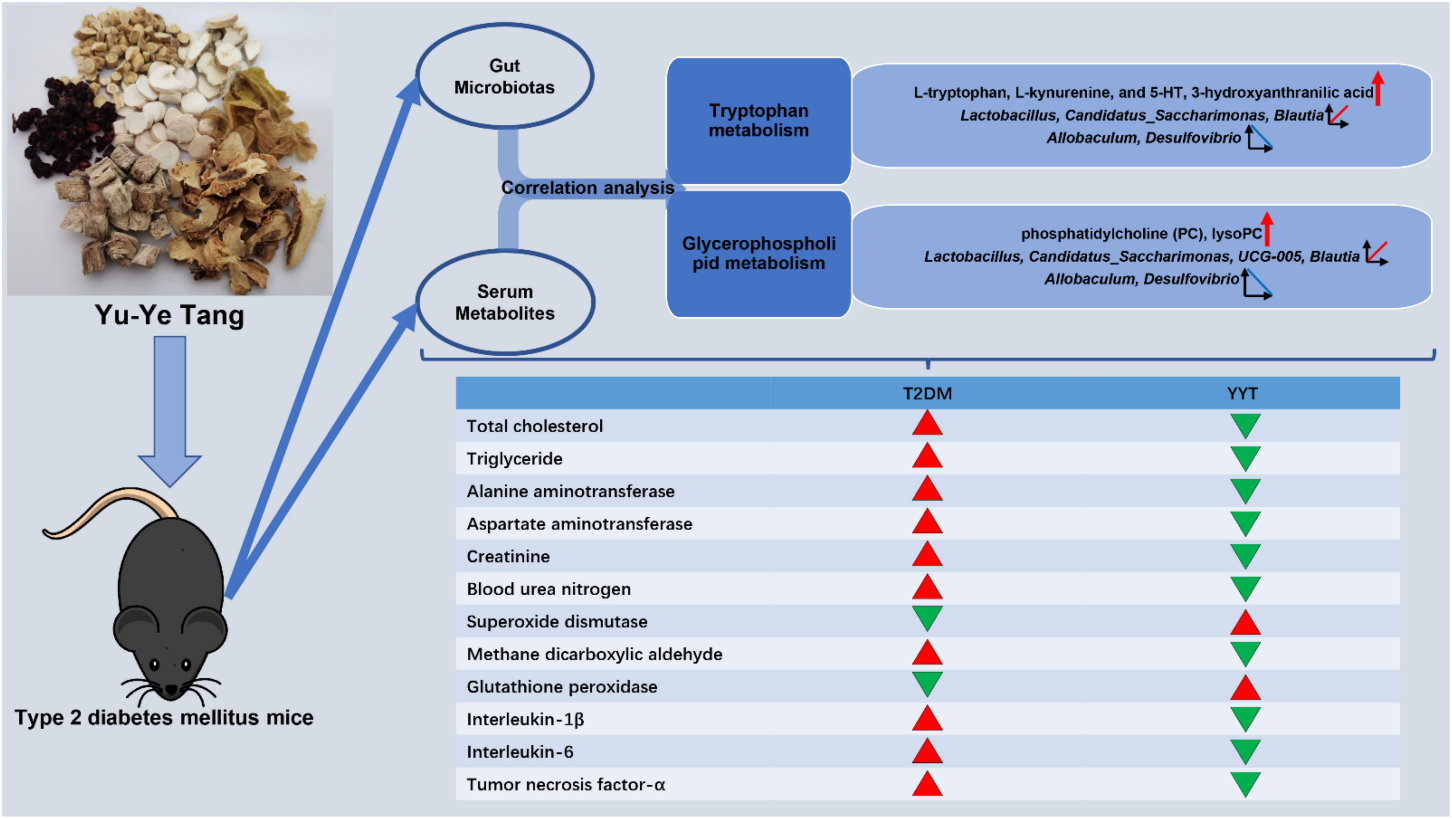
Graphical abstract.

This study revealed that YYT has a wide range of therapeutic effects on T2DM rats, including antioxidative and anti-inflammatory effects. Furthermore, YYT corrected the altered gut microbiota and serum metabolites in T2DM rats. This study suggests that YYT may have a therapeutic impact on T2DM by regulating gut microbiota and modulating tryptophan and glycerophospholipid metabolism, which are potential key pathways in treating T2DM (**Figure 8**).

## Data availability statement

The sequencing data presented in the study are deposited in the Sequence Read Archive repository, accession number PRJNA931818.

## Ethics statement

The animal study was approved by ethics committee of Cangzhou Integrated Traditional Chinese and Western Medicine Hospital. The study was conducted in accordance with the local legislation and institutional requirements.

## Author contributions

ZM and WS carried out the experiments and manuscript writing. LW, YW, and BP provided experimental help. XS, HL, and HZ performed data analysis and result interpretation. SL provided ideas and technical guidance for the whole work. HW supervised the experiments. All authors have read and agreed to the published version of the manuscript.
