# Supplementary material for: Integrated 16S rRNA sequencing and nontargeted metabolomics analysis to reveal the mechanisms of Yu-Ye Tang on type 2 diabetes mellitus rats
Source: Front Endocrinol (Lausanne). 2023 Sep 5;14:1159707. doi: 10.3389/fendo.2023.1159707 (PMC10507721; doi:10.3389/fendo.2023.1159707)
Supplement: Supplementary file 1 [file DataSheet_1.docx]

**HPLC analysis of YYT**

As previously reported, puerarin (C_21_H_20_O_9_, CAS: 3681-99-0), mangiferin (C_19_H_18_O_11_, CAS: 4773-96-0) and calycosin 7- O-β-D-glucopyranoside (C_22_H_22_O_10_, CAS: 20633-67-4) were considered as the main symbolic compounds of YYT (1). Therefore, these three compounds were selected for quality control of YYT in this study. The specific methods were as follows. YYT was dissolved in equal volume of 80% methanol. Then, the solution was filtered with a 0.22 μm filter membrane. Then, high performance liquid chromatography (HPLC) with an ultraviolet detector (Aligent, LC 1220, US) was used to identify the major compounds in YYT. Agilent Zorbax SB-C18 column (4.6 mm × 250 mm, 5 μm) was used for gradient elution with methanol as the mobile phase A and 0.2% formic acid solution as the mobile phase B. The flow rate was 1.0 mL/min, the detection wavelength was 260 nm and the column temperature was 35 ℃. The gradient elution mode was as follows: 0–20 min (20–40% A); 20–25 min (40% A). It was found that the characteristic spectra of YYT were consistent with those of the standards, proving that YYT had been correctly prepared (**Fig. S1**).

**Figure S1 The HPLC analysis of YYT**


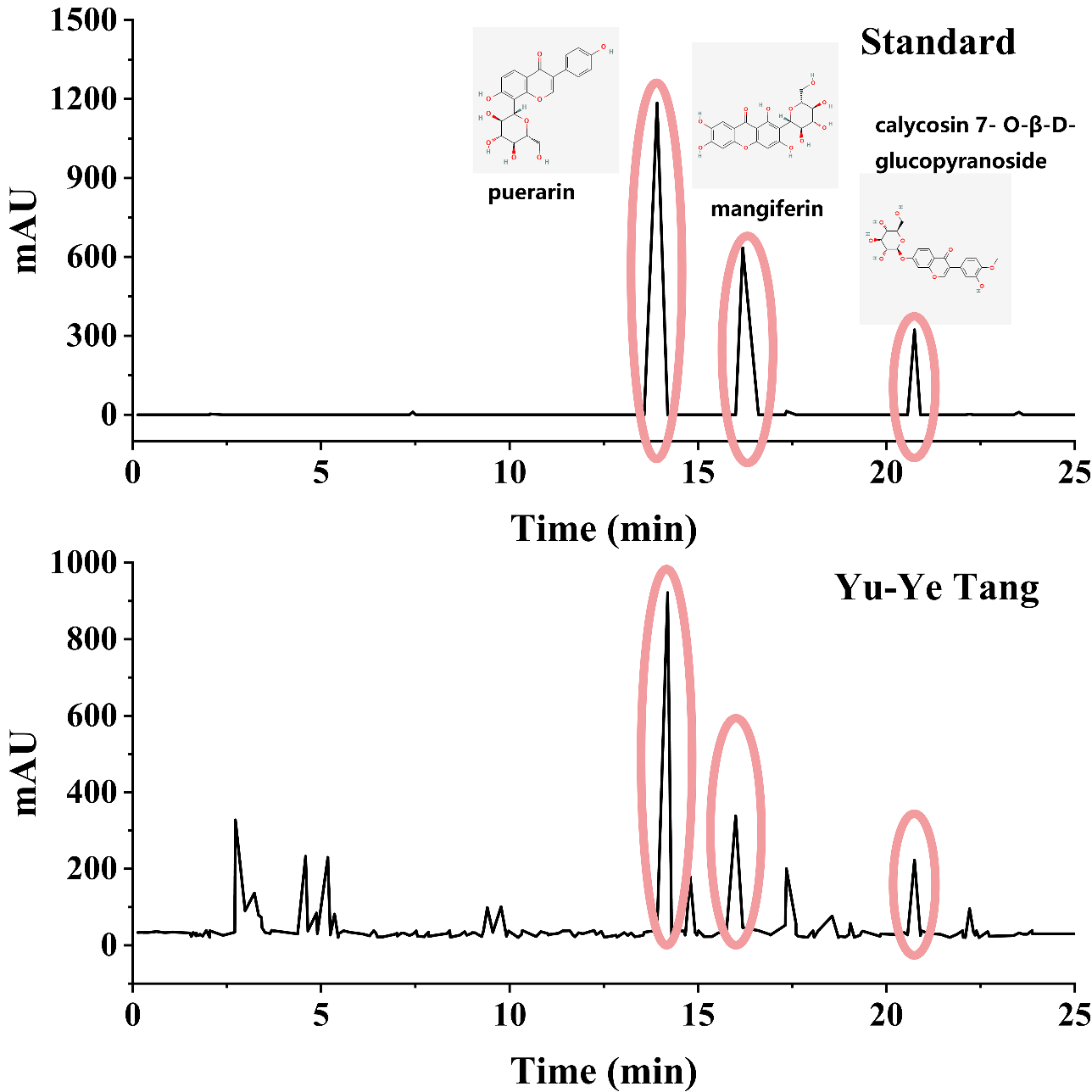


**Detailed methods for 16S rRNA gene sequencing**

**Polymerase chain reaction (PCR) amplification and 16S rRNA gene sequencing**

Based on the characteristics of the amplified 16S region, PCR amplification of the hypervariable region of 16S rDNAV3-V4 was performed using the amplification primers 338F (5′-ACTCCTACGGGAGGCAGCAG-3′) and 806R (5′-GGACTACHVGGGTWTCTAAT-3′). After performing 2% agarose gel electrophoresis to quantify the amplified products, the Illumina NovaSeq platform was used for sequencing, and a 250 bp paired-end sequence was obtained. After the raw data were obtained by sequencing, they were assembled and filtered to obtain the effective tags. Subsequently, the UParse software (Uparsev 7. 0. 1001) was used to cluster the effective tags of all samples, and the sequences with 97% sequence similarity were clustered into operational taxonomic units (OTUs).

**Sequencing data processing and analysis**

Diversity analysis was performed on the sequencing data results. Shannon and Simpson indices were used to assess the gut microbiota alpha diversity of each group. The results of beta diversity analysis were presented using principal coordinate analysis (PCoA) plots, an unconstrained data downscaling analysis that presents similarities and differences in community composition across sample groups. The Wilcoxon rank–sum test was used to test for differences between groups in diversity indices. In addition, OTU was used for species classification of OTU by comparing with the database, and the species abundance of each group of samples was analyzed at the phylum and genus levels. At genus level, 27 differential taxa between control and model group, and there are 12 differential taxa between model and YYT-H group were screened. The taxa that are both differential between control and model group and between model and YYT-H group are screened in this manuscript.Finally, the analysis of Phylogenetic Investigation of Communities by Reconstruction of Unobserved States database (PICRUSt) was used to predict the relevant gene pathways that may be affected by each group of differential microbiotas.

**Detailed methods for nontargeted metabolomics analysis**

An equal amount of each sample was obtained and mixed to be used as quality control samples. Periodic analysis was performed throughout the process to monitor the stability of the instrument. Nontargeted metabolomic analysis was performed using liquid chromatography–mass spectrometry (LC-MS) based on our previous studies.

High-resolution mass spectrometry (HRMS) detection was employed, and peak identification, retention time correction, automatic integration, and other preprocessing steps were also performed based on mzCloud (<https://www.mzcloud.org/>), mzVault, and MassList databases and Compound Discoverer 3.1 (CD3.1, Thermo Fisher) software. The missing values of metabolomics data were proceed as follows, 1) Calculates the detection limit for each missing ion as an area of a simulated Gaussian peak that starts and ends at a zero intensity baseline. To calculate the area of the Gaussian peak, the node uses the expected peak width and the maximum spectrum noise in the expected retention time range multiplied by the S/N threshold; 2) Searches for the missing ion with the expected m/z × RT dimensions against all detected ions (in the mass list generated by the Detect Compounds node) while ignoring the assigned adduct type. If it finds a match (ion with the expected m/z × RT dimensions), it uses the ion’s area to fill the gap and displays Filled by Matching Ion for the Fill Status; 3) If the node does not find a matching ion, it attempts to detect the peak at a lower intensity threshold using the Parameterless Peak Detection (PPD) algorithm. If it detects a chromatographic peak at a lower threshold, it uses the integrated peak area to fill the gap and displays Re-detected Peak for the Fill Status; 4) If the node does not find a chromatographic peak by using a lower intensity threshold, it fits a Gaussian peak to the XIC trace for the expected m/z range and displays Filled by Simulated Peak for the Fill Status; 5) If the filled area is still zero or lower than the detection limit, the node uses the detection limit value to fill the gap and displays Filled by Spectrum Noise for the Fill Status; 6) If the node cannot fill the gap, it displays Area Could Not Be Filled for the Fill Status.

Then, a visualizable matrix containing metabolite number, retention time, m/z, metabolite name, ion pattern, peak area, etc., was obtained, in which the area of each characteristic peak represented the relative quantitative value of a metabolite. After the quantitative results were normalized using the total peak area, the quantitative results of the metabolite were obtained. The intragroup similarity and intergroup variability of the three sample groups were analyzed using score plots of principal component analysis (PCA) and partial least squares-discriminant analysis (PLS-DA). Furthermore, the metabolites that differed significantly and contributed highly in the two groups of samples (control and T2DM groups, YYT-L and T2DM groups) were obtained from the PLS-DA score plot. Metabolites that differed significantly between the groups were screened based on variable importance in projection (VIP) > 1, *p* ˂ 0.05, and fold change (FC) ≥ 1.2 or ≤ 0.8. A total of 1305 metabolites were identified from the LC-MS results. A total of 493 metabolite features in negative ion mode and 812 metabolite features in positive ion mode.Statistical analysis and metabolic pathway enrichment analysis of differential metabolites were performed using MetaboAnalyst. Differential pathways were screened according to variable impact value > 1 and *t*-test *p* < 0.05.

Reference

| 1. | Guo F, Yao L, Zhang W, Chen P, Hao R, Huang X, Jiang J, Wu S. The therapeutic mechanism of Yuye decoction on type 2 diabetes mellitus based on network pharmacology and experimental verification. *Journal of Ethnopharmacology* (2023) **308**:116222. doi:10.1016/j.jep.2023.116222 |
| --- | --- |
